# Associations between blood coagulation markers, NT-proBNP and risk of incident heart failure in older men: The British Regional Heart Study

**DOI:** 10.1016/j.ijcard.2016.12.056

**Published:** 2017-03-01

**Authors:** S. Goya Wannamethee, Peter H. Whincup, Olia Papacosta, Lucy Lennon, Gordon D. Lowe

**Affiliations:** a Department of Primary Care and Population Health, University College London, UK; b Population Health Research Centre, Division of Population Health Sciences and Education, St George's, University of London, UK; c Institute of Cardiovascular & Medical Sciences, University of Glasgow, UK

**Keywords:** Coagulation, D-dimer, NT-proBNP, Heart failure

## Abstract

**Aims:**

Chronic heart failure (HF) is associated with activation of blood coagulation but there is a lack of prospective studies on the association between coagulation markers and incident HF in general populations. We have examined the association between the coagulation markers fibrinogen, von Willebrand Factor (VWF), Factors VII, VIII and IX, D-dimer, activated protein C (APC) and activated partial thromboplastin time (aPPT) with NT-proBNP and incident HF.

**Methods and results:**

Prospective study of 3366 men aged 60–79 years with no prevalent HF, myocardial infarction or venous thrombosis and who were not on warfarin, followed up for a mean period of 13 years, in whom there were 203 incident HF cases. D-dimer and vWF were significantly and positively associated with NT-proBNP (a marker of neurohormonal activation and left ventricular wall stress) even after adjustment for age, lifestyle characteristics, renal dysfunction, atrial fibrillation (AF) and inflammation (C-reactive protein). By contrast Factor VII related inversely to AF and NT-proBNP even after adjustment. No association was seen however between the coagulation markers VWF, Factor VII, Factor VIII, Factor IX, D-dimer, APC resistance or aPPT with incident HF in age-adjusted analyses. Fibrinogen was associated with incident HF but this was abolished after adjustment for HF risk factors.

**Conclusion:**

Coagulation activity is not associated with the development of HF. However D-dimer and vWF were significantly associated with NT-proBNP, suggesting that increased coagulation activity is related to cardiac stress; and the increased coagulation seen in HF patients may in part be a consequence of neurohormonal activation.

## 1 Introduction

It is well established that patients with chronic heart failure (HF) have systemic activation of blood coagulation, which may partly reflect systemic activation of inflammation, and which may increase their risk of arterial and venous thromboembolic events and with adverse prognosis [1], [2], [3], [4]. Such patients have increased levels of fibrinogen and the Factor VIII – von Willebrand factor (VWF) complex; and elevated levels of coagulation activation markers including fibrin D-dimer [1], [2], [3], [4]. Plasma levels of fibrinogen and VWF are associated with adverse prognosis in such patients [5]. Moreover fibrinogen and vWF-VIII have been associated with incident atrial fibrillation (AF), a major risk factor for HF [6]. While it is well established that fibrinogen [7], VWF-VIII [8] and D-dimer [8] are associated with increased risk of coronary heart disease (CHD), there is a lack of prospective studies of coagulation markers and risk of incident HF.

In the British Regional Heart Study (BRHS), we have assayed a range of coagulation markers in men aged 60–79 years, and have previously reported associations of fibrinogen, VWF, Factors VIII and IX, and D-dimer with incident myocardial infarction (MI) and CHD death [9]. No significant associations in this report were observed for Factors VII, activated partial thromboplastin time (aPTT) or activated protein C (APC) resistance, as measured by a low APC ratio [10]. To assess the possible role of coagulation activation in pathogenesis of HF, we have now examined the associations of these coagulation markers with incident HF, in those men who had no baseline diagnosis of HF or arterial or venous thrombosis or who were taking warfarin. We also examined their associations with baseline levels of NT-proBNP, and with the presence of atrial fibrillation at baseline, to investigate associations with cardiac stress.

## 2 Subjects and methods

The British Regional Heart Study is a prospective study of cardiovascular disease involving 7735 men aged 40–59 years drawn from general practice in each of 24 British towns, who were screened between 1978 and 1980 [11]. The population studied was socio-economically representative of British men but consisted almost entirely of white Europeans (> 99%). In 1998–2000, all surviving men, now aged 60–79 years (mean age 68.7 years), were invited for a 20th year follow-up examination. The men completed a questionnaire which included questions on their medical history and lifestyle behaviour. They were requested to fast for a minimum of 6 h, during which time they were instructed to drink only water and to attend for measurement at a pre-specified time between 0800 and 1800 h. All men were asked to provide a blood sample, collected using the Sarstedt Monovette system. 4252 men (77% of survivors) attended for examination. 4088 men had at least one haemostatic/inflammatory marker measured. We excluded men with doctor diagnosed MI or a recall of a diagnosis of deep vein thrombosis or pulmonary embolism and men on warfarin (*n* = 722). After these exclusions 3366 men were available for analysis.

### 2.1 Cardiovascular risk factor measurements at 1998–2000

Anthropometric measurements including body weight, height and waist circumference (WC) were carried. Details of measurement and classification methods for smoking status, physical activity, social class, alcohol intake, blood pressure and blood lipids in this cohort have been described [12], [13]. Predicted glomerular filtration rate (eGFR) (measure of renal function) was estimated from serum creatinine using the equation eGFR = 186 x (creatinine)-1.154 x (age)-0.203 [14]. Renal dysfunction was defined as eGFR < 60. N-terminal pro-brain natriuretic peptide (NT-proBNP) was determined using the Elecsys 2010 electrochemiluminescence method (Roche Diagnostics, Burgess Hill, UK) [15]. Electrocardiographic LVH was defined according to relevant Minnesota codes (codes 3.1 or 3.3). Atrial fibrillation (AF) was defined according to Minnesota codes 8.3.1 and 8.3.3.

### 2.2 Haemostatic and inflammatory markers

At the 20-year examination, blood was anticoagulated with 0.109 M trisodium citrate (9:1 v:v) for measurement of clottable fibrinogen (Clauss method); as well as coagulation factors VII, VIII and IX; activated partial thromboplastin time (APTT) and activated protein C (APC) ratio (measured by the aPTT-based method) in an MDA-180 coagulometer (Organon Teknika, Cambridge, UK). Plasma levels of D-dimer were measured with enzyme-linked immunosorbent assays (Biopool AB, Umea, Sweden) as was von Willebrand factor (VWF) antigen (DAKO, High Wycombe, UK). C-reactive protein was assayed by ultra-sensitive nephelometry (Dade Behring, Milton Keynes, UK). Distributions and laboratory coefficients of variation have been described previously [9].

### 2.3 Follow-up

All men have been followed up from initial examination (1978–1980) for cardiovascular morbidity [16] and follow-up has been achieved for 99% of the cohort. In the present analyses, all-cause mortality and morbidity events are based on follow-up from re-examination in 1998–2000 at mean age 60–79 years to June 2012. Survival times ended at the first heart failure event or when they were censored for death due to any cause, or the end of the follow-up period (June 2012), whichever occurred first. Information on death was provided by the UK National Health Service registers. Fatal CHD events were defined as death with CHD (ICD 9th revision, codes 410–414) as the underlying code. A non-fatal MI was diagnosed according to World Health Organisation criteria. Evidence of non-fatal MI and HF was obtained by ad hoc reports from general practitioners supplemented by biennial reviews of the patients' practice records (including hospital and clinic correspondence) through to the end of the study period. Incident CHD included fatal CHD and non-fatal MI. Incident non-fatal HF was based on a doctor-confirmed diagnosis of HF from primary care medical records (including hospital and clinical correspondence) [15]. All cases were verified by a review of available clinical information from primary and secondary care records (symptoms, signs, investigations, and treatment response) to ensure they are consistent with current recommendations on HF diagnosis [17]. Incident fatal HF cases were those in which the diagnosis of HF was mentioned as the underlying cause of death at death certificates (ICD 9th revision code 428). Incident HF included both incident non-fatal HF and incident fatal HF.

### 2.4 Statistical methods

Analyses are based on the division of haemostatic markers into equal quartiles. Cox's proportional hazards model was used to assess the multivariate-adjusted relative risk for the highest quarter compared with the lowest quarter (reference group). In multivariate analyses, smoking (never, long term ex-smokers (> 15 years), recent ex-smokers (< 15 years) and current smokers), social class (manual vs non manual), physical activity (4 groups, diabetes (yes/no), use of antihypertensive treatment (yes/no), renal dysfunction (yes/no), LVH (yes/no) and AF (yes/no) were fitted as categorical variables. Systolic blood pressure, HDL-C and CRP were fitted as continuous variables. Tests for trends were carried out fitting the haemostatic markers in its original continuous form. All analyses were performed with SAS version 9.3 (SAS, Cary, North Carolina).

## 3 Results

During the mean follow-up period of 13 years there were 203 incident heart failure events in the 3366 men with no prevalent HF, arterial or venous thrombosis and who were not on warfarin, a rate of 5.5/1000 person years. Table 1 shows baseline characteristics in the study population by incident HF status. Table 2 shows the association between the coagulation markers and prevalence of AF and mean NT-proBNP. Elevated D-dimer and vWF were significantly associated with higher prevalence of AF, but this was attenuated after adjustment for age (*p* = 0.10 for trend for both variables). However, both markers were significantly associated with NT-proBNP, even after adjustment for established risk factors and inflammation (Table 2). In contrast Factor VII was inversely associated with both AF and NT-proBNP even after adjustment for HF risk factors in Table 2. The adjusted odds ratio of having AF in those with elevated Factor VII (top quartile) compared to those in the bottom quartile was 0.27 (0.13,0.56) (*p* < 0.0001). The inverse association between Factor VII and AF remained even after additional adjustment for NT-proBNP (*p* = 0.02). Factor IX showed a significant inverse association with NT-proBNP, but only after adjustment.

We also examined the association between levels of NT-proBNP and coagulation markers D-dimer, vWF and Factor VII to see whether there is a threshold effect of NT-proBNP (Table 3). D-dimer and vWF increased progressively with increasing NT-proBNP even after adjustment. By contrast Factor VII decreased progressively with increasing NT-proBNP.

Table 4 shows the association between the 8 coagulation markers and risk of incident HF. With the exception of fibrinogen, no significant association was seen between coagulation markers and incident HF in age-adjusted analysis; and the association with fibrinogen was abolished after adjustment for risk factors for HF. Factor VII was inversely associated with incident HF only after adjustment for HF risk factors but this was largely associated with NT-proBNP.

## 4 Discussion

In this study of older British men with no prevalent HF or arterial or venous thrombosis and who were not on warfarin, coagulation markers including fibrinogen, D-dimer, vWF, factors VII, VIII, IX, APC ratio and APPT were not associated with incident HF after adjustment for age and established HF risk factors. However, D-dimer and vWF were significantly associated with NT-proBNP, a marker of neurohormonal activation and left ventricular wall stress and a strong predictor of HF. However, Factor VII was inversely associated with cardiac stress (AF and NT-proBNP) even after adjustment. This is the first comprehensive prospective study of coagulation markers and incident HF.

### 4.1 Coagulation activation and incident heart failure

Although D-dimer has been strongly associated with CHD and stroke in this study [9], [18], [19], no association was seen between D-dimer and incident HF. Similarly levels of vWF and Factor VIII related to incident CHD, but not to HF. Fibrinogen was associated with HF in age-adjusted analyses. This association was abolished after adjustment for other established risk factors for HF. Although clinical studies have shown activated protein C to have anti-inflammatory and anti-apoptotic effects [20] and animal models have shown treatment with APC to protect the development of myocardial fibrosis which contributes to the development of HF [21] no association was seen between APC resistance (APC ratio) and risk of incident HF. Factor VII related inversely to incident HF, which was only significant in the multivariate analysis; no significant association was seen in the age-adjusted analyses. The lack of association between all 8 coagulation markers studied and risk of incident HF suggests that coagulation does not play a role in the development of HF at least in older adults. This suggests that, in contrast to coronary heart disease events which may have thromboembolic mechanisms, heart failure in older adults is more related to other pathophysiological mechanisms. This is in keeping with the findings that older patients with HF tend to have preserved ejection fraction [22]. In addition, these patients are less likely to have CHD and more likely to have hypertension and atrial fibrillation [23]. This is consistent with the findings of our own study, in which a high proportion of men without prior MI who went on to develop HF did not develop MI before developing HF (85%).

### 4.2 Coagulation activation and baseline cardiac stress (NT-proBNP)

Chronic heart failure is increasingly recognised as a syndrome which confers a considerable prothrombotic risk [24], [25]. The pathogenesis of increased coagulation activation in such patients is complex and not fully understood. Neurohormonal activation has been hypothesised to be a prothrombotic mechanisms in patients with HF [24], [25]. In HF increased wall stretch, neurohormonal activation and hypoxia stimulate BNP secretion [26]. In this general population study, we have shown a significant association between NT-proBNP and D-dimer and vWF independent of other known factors associated with NT-proBNP, including inflammation, in subjects free of HF. Levels of D-dimer and vWF increased progressively with increasing levels of NT-proBNP. The findings of an association between coagulation activation with NT-proBNP but not incident HF suggests that raised D-dimer and vWF in those with HF is likely to be a consequence of neurohormonal activation. By contrast we did not observe an association between vWF and D-dimer with baseline AF after adjustment for age. With the exception of Factor VII no other coagulation markers related to AF.

### 4.3 Factor VII and cardiac stress and incident HF

An unexpected finding in the study was the inverse association seen between Factor VII and NT-proBNP and AF and incident HF after adjustment for established HF risk factors. The inverse association between Factor VII and incident HF was however largely due to the inverse association with NT-proBNP. The inverse association seen between Factor VII and NT-proBNP may explain why low plasma concentrations of coagulation factor VII has been linked with increased of CVD mortality in elderly patients with symptoms of HF [27]. The nature of the inverse association between Factor VII and cardiac stress is unclear and warrants further investigation in other studies.

### 4.4 Atrial fibrillation in heart failure patients

Heart failure is common in patients with AF; these patients have been shown to have significantly higher 1-year mortality and morbidity rates (resulting from a combination of stroke,thrombo-embolism and transient ischaemic attacks) than AF patients without heart failure, despite high rates of oral anticoagulant use [28]. We did not find AF to be associated with coagulation markers except inversely with factor VII. In contrast, NT-proBNP (which is raised in heart failure) was significantly associated with vWF and D-dimer, factors shown to be associated with increased risk of stroke [19] and overall mortality. Thus, neurohormonal activation in heart failure may contribute to the increased risk of stroke seen in AF patients with heart failure.

#### 4.4.1 Strengths and limitations

We have included a comprehensive evaluation of coagulation markers which have been related to other CVD outcomes including CHD and stroke [9], [19]. However, the findings are based on an older predominantly white male population of European extraction, so that the results cannot be generalized directly to women, younger populations or other ethnic groups. The current findings are based on doctor diagnosed HF, which is likely to underestimate the true incidence of HF in this study population. It is also possible that there is survival bias in our data in that men who attended the re-examination were healthier than those who did not; this may account for the null associations seen with prevalent AF. Although survivor bias may have affected the absolute incidence rate of HF, this should not have affected the associations between coagulation markers and incident HF. The determinants of HF in this study population (including obesity and NT-proBNP) [12], [15] generally accord with prior data and therefore suggest potential external validity for our findings. We had no information on incident AF. Information on echocardiogram measurements was not available in all men and we were not therefore able to differentiate systolic and diastolic HF.

### 4.5 Conclusion

We have shown in a large prospective study of older British men that the coagulation markers D-dimer and vWF are associated with baseline evidence of cardiac stress (NT-proBNP) but not with incident HF. We conclude that coagulation activation in men at risk of HF may be a consequence of neurohormonal activation and is not of causal significance in the development of HF. If so, future approaches for reducing the thrombotic state in persons with CHF include pharmacological modulation of neurohormonal activation. The unexpected finding of an inverse association between Factor VII and cardiac stress requires further investigation.

## Conflict of interest

None.

## Author contributions

SGW initiated the concept and design of the paper, analysed the data and drafted the manuscript. PHW and GDL contributed to the interpretation of data. OP contributed to the analysis of the paper. LL and PHW contributed to the acquisition of the data. All authors revised it critically for important intellectual content and approved the final version of the manuscript.

## Funding

This work was supported by the British Heart Foundation Programme grant RG/13/16/30528.

## Figures and Tables

**Table 1:** Baseline characteristics (1998–2000) in 3336 men without prevalent diagnosed myocardial infarction, venous thrombosis or Heart Failure and who were not on warfarin according to incident Heart Failure status.

	No HF (*N* = 3163)	Developed HF (*N* = 203)
Age (yrs)	68.28 (5.45)	70.64 (5.45)
BMI (kg/m^2^)	26.73 (3.58)	27.53 (3.37)
% inactive	33.5	37.0
% current smokers	12.9	12.3
% manual	53.2	56.7
% heavy drinkers	3.7	6.4
% on antihypertensive drugs	27.0	42.4
% stroke	4.1	4.4
% diabetes	5.7	5.9
% renal dysfunction	13.6	22.2
% atrial fibrillation	1.9	7.4
% LVH	7.2	14.3
SBP (mm Hg)	149.9 (23.8)	156.0 (23.0)
Cholesterol (mmol/L)	6.05 (1.05)	5.91 (1.04)
HDL-cholesterol (mmol/L)	1.33 (0.34)	1.33 (0.36)
CRP (mg/L)*	1.63 (0.79–3.23)	2.14 (1.03–3.68)
NT-proBNP (ng/L)	80.6 (41–152)	190.6 (81.5–405.5)
Fibrinogen (g/L)	3.24 (0.72)	3.38 (0.77)
D-dimer (μg/L)*	77.6 (48–122)	90.9 (51–127)
vWF (IU/L)	1369 (451)	1426 (482)
FVII (IU/L)	1199 (226)	1188 (268)
FVIII (IU/L)	1310 (314)	1353 (309)
FIX (IU/L)	1332 (226)	1347 (231)
APPT (s)	30.78 (3.42)	30.75 (2.54)
APC ratio	3.26 (0.50)	3.27 (0.52)

Mean (SD) unless specified; *geometric mean and interquartile range.

**Table 2:** Markers of coagulation and associations with prevalent AF and baseline mean NT-proBNP (ng/L) in men without prevalent diagnosed myocardial infarction, venous thrombosis or heart failure and who were not on warfarin.

		Quartiles			
	1	2	3	4	*p-Trend*
*D-dimer*					
% AF	1.2	1.7	1.9	4.0	*0.0004*
NT-proBNP	58.0	79.5	90.8	124.6	*< 0.0001*
*Adjusted	73.7	83.1	83.9	98.5	*< 0.0001*

*Fibrinogen*					
% AF	1.4	2.2	2.1	3.2	*0.10*
NT-proBNP	70.1	80.8	84.0	109.8	*< 0.0001*
Adjusted	86.5	85.6	80.6	86.5	*< 0.50*

*vWF*					
% AF	1.6	1.8	2.7	2.8	*0.18*
NT-proBNP	70.1	75.8	86.4	113.0	*< 0.0001*
Adjusted	81.5	79.8	83.9	93.7	*0.008*

*Factor VII*					
% AF	4.0	2.9	0.9	1.3	*< 0.0001*
NT-proBNP	103.5	87.4	77.5	75.9	*< 0.0001*
Adjusted	107.8	85.6	79.0	72.2	*< 0.0001*

*Factor VIII*					
% AF	1.5	1.4	3.1	3.1	*0.02*
NT-proBNP	71.1	78.9	89.6	100.8	*< 0.0001*
Adjusted	83.1	82.3	87.4	85.6	*0.60*

*FACTOR IX*					
% AF	2.4	1.9	1.4	3.1	*0.10*
NT-proBNP	91.0	83.0	81.4	85.0	*0.12*
Adjusted	96.5	89.1	79.0	75.2	*< 0.0001*

*APPT*					
% AF	2.2	1.6	2.7	2.4	*0.52*
NT-proBNP	83.4	80.3	83.9	92.7	*0.03*
Adjusted	84.8	82.3	81.5	90.0	*0.20*

*APC ratio*					
% AF	2.1	1.4	2.1	3.3	*0.07*
NT-proBNP	76.7	82.3	87.4	95.1	*0.002*
Adjusted	79.8	82.3	84.8	90.0	*0.09*

⁎ Adjusted for age, smoking, BMI, use of antihypertensive treatment, physical activity, systolic blood pressure, LVH, prevalent stroke, renal dysfunction, heavy drinking, CRP and AF.

**Table 3:** Associations between levels of NT-proBNP and D-dimer and vWF in men without prevalent diagnosed myocardial infarction, venous thrombosis or Heart Failure and who were not on warfarin.

			NT-proBNP (ng/L)			
	< 50	50–99	100–149	150–299	300	
	*N* = 966	*N* = 886	*N* = 437	*N* = 495	*N* = 354	
*D-dimer (μg/L)*						
Adjusted mean	74.4	79.0	83.1	90.0	93.7	< 0.0001
Adjusted odds elevated D-dimer	1.00	1.18 (0.92,1.51)	1.47 (1.10,1.98)	1.64 (1.23,2.17)	1.78 (1.27,2.49)	< 0.0001

*vWF (IU/L)*						
Adjusted mean	1327	1360	1377	1400	1435	< 0.0001
Adjusted odds elevated vWF	1.00	1.25 (0.98,1.58)	1.28 (0.96,1.71)	1.51 (1.14,2.00)	1.65 (1.19,2.29)	0.0008

*Factor VII (IU/L)*						
Adjusted mean	1237	1203	1213	1165	1129	< 0.0001
Adjusted odds elevated Factor VII	1.00	0.82 (0.66,1.01)	0.90 (0.69,1.17)	0.59 (0.45,0.78)	0.38 (0.27,0.54)	< 0.0001

*Adjusted for age, smoking, BMI, use of antihypertensive treatment, physical activity, systolic blood pressure, LVH, prevalent stroke, renal dysfunction, heavy drinking, AF and CRP.

Elevated = top tertile.

**Table 4:** Markers of coagulation and risk of HF in men with no prevalent MI, HF or DVT/PE and who are not on warfarin.

		Quartiles			
	1	2	3	4	*p-trend*
*D-Dimer*					
Rates/1000 p-yrs	4.4	5.7	5.5	6.7	
Age-adjusted	1.00	1.06 (0.71,1.59)	0.91 (0.60,1.37)	1.02 (0.67,1.54)	0.18
Model 1	1.00	0.94 (0.62,1.41)	0.76 (0.49,1.17)	0.80 (0.52,1.25)	0.69

*Fibrinogen*					
Rates/1000 p-yrs	3.3	5.7	6.0	6.8	
Age-adjusted	1.00	0.95 (0.62,1.45)	1.26 (0.85,1.87)	1.37 (0.92,2.03)	0.01
Model 1	1.00	0.87 (0.56,1.34)	1.13 (0.74,1.72)	1.05 (0.65,1.68)	0.35

*vWF*					
Rates/1000 p-yrs	4.2	5.6	6.0	6.5	
Age-adjusted	1.00	1.23 (0.82,1.84)	1.24 (0.82,1.86)	1.22 (0.81,1.83)	0.23
Model 1	1.00	1.20 (0.79,1.82)	1.13 (0.74,1.72)	1.06 (0.69,1.62)	0.79

*Factor VII*					
Rates/1000 p-yrs	5.9	6.5	5.2	4.6	
Age adjusted	1.00	1.01 (0.70,1.47)	0.85 (0.57,1.25)	0.75 (0.50,1.12)	0.47
Model 1	1.00	0.97 (0.66,1.42)	0.79 (0.53,1.17)	0.64 (0.42,0.96)	0.07
Model 1 + NT-proBNP	1.00	1.04 (0.70,1.54)	0.90 (0.60,1.36)	0.80 (0.52,1.21)	0.63

*Factor VIII*					
Rates/1000 p-yrs	3.8	5.6	6.0	6.8	
Age adjusted	1.00	1.41 (0.93,2.14)	1.44 (0.94,2.20)	1.46 (0.96,2.22)	0.11
Model 1	1.00	1.42 (0.93,2.16)	1.29 (0.83,1.99)	1.28(0.82,1.98)	0.53

*Factor IX*					
Rates/1000 p-yrs	4.3	5.4	6.9	5.6	
Age-adjusted	1.00	1.24 (0.82,1.88)	1.52 (1.02,2.27)	1.28 (0.84,1.94)	0.17
Model 1	1.00	1.18 (0.77,1.80)	1.33 (0.88,2.01)	0.93 (0.59,1.46)	0.73

*APPT*					
Rates/1000 p-yrs	5.6	5.2	5.5	5.8	
Age-adjusted	1.00	0.93 (0.63,1.38)	1.00 (0.69,1.46)	1.05 (0.71,1.55)	0.85
Model 1	1.00	1.00 (0.67,1.49)	1.02 (0.69,1.49)	1.03 (0.69,1.54)	0.88

*APC ratio*					
Rates/1000 p-yrs	4.8	5.5	5.8	6.1	
Age-adjusted	1.00	1.07 (0.72,1.58)	1.10 (0.74,1.63)	1.20 (0.81,1.71)	0.76
Model 1	1.00	1.09 (0.73,1.62)	1.05 (0.70,1.59)	1.06 (0.71,1.64)	0.73

Model 1 adjusted for age, smoking, BMI, use of antihypertensive treatment, physical activity, systolic blood pressure, LVH, prevalent stroke, renal dysfunction, heavy drinking, CRP.
